# Automatic biometry of fetal brain MRIs using deep and machine learning techniques

**DOI:** 10.1038/s41598-023-43867-4

**Published:** 2023-10-19

**Authors:** Jiayan She, Haiying Huang, Zhijun Ye, Wei Huang, Yan Sun, Chuan Liu, Weilin Yang, Jiaxi Wang, Pengfei Ye, Lei Zhang, Gang Ning

**Affiliations:** 1 Key Laboratory of Birth Defects and Related Diseases of Women and Children, Ministry of Education, Department of Radiology, West China Second University Hospital, Sichuan University, Chengdu, 610041 China; 2 Machine Intelligence Laboratory, College of Computer Science, Sichuan University, Chengdu, 610065 People’s Republic of China

**Keywords:** Computer science, Medical research, Paediatric research

## Abstract

Linear biometric measurements on magnetic resonance images are important for the assessment of fetal brain development, which is expert knowledge dependent and laborious. This study aims to construct a segmentation-based method for automatic two-dimensional biometric measurements of fetal brain on magnetic resonance images that provides a fast and accurate measurement of fetal brain. A total of 268 volumes (5360 images) magnetic resonance images of normal fetuses were included. The automatic method involves two steps. First, the fetal brain was segmented into four parts with a deep segmentation network: cerebrum, cerebellum, and left and right lateral ventricles. Second, the measurement plane was determined, and the corresponding biometric parameters were calculated according to clinical guidelines, including cerebral biparietal diameter (CBPD), transverse cerebellar diameter (TCD), left and right atrial diameter (LAD/RAD). Pearson correlation coefficient and Bland–Altman plots were used to assess the correlation and agreement between computer-predicted values and manual measurements. Mean differences were used to evaluate the errors quantitatively. Analysis of fetal cerebral growth based on the automatic measurements was also displayed. The experiment results show that correlation coefficients for CBPD, TCD, LAD and RAD were as follows: 0.977, 0.990, 0.817, 0.719, mean differences were − 2.405 mm, − 0.008 mm, − 0.33 mm, − 0.213 mm, respectively. The correlation between the errors and gestational age was not statistically significant (p values were 0.2595, 0.0510, 0.1995, and 0.0609, respectively). The proposed automatic method for linear measurements on fetal brain MRI achieves excellent performance, which is expected to be applied in clinical practice and be helpful for prenatal diagnosis and clinical work efficiency improvement.

## Introduction

Assessment of fetal brain development is important for prenatal diagnosis. Fetal brain biometry, which is a quantitative evaluation of the fetal brain and its various anatomical segmentations using two-dimensional (2D) and three-dimensional (3D) measurements, is an important part of prenatal screening for central nervous system malformations because it contributes to the determination of gestational age (GA), fetal weight estimation, fetal growth and development monitoring, and abnormality diagnosis and prognosis prediction^1–4^. Among them, 2D linear measurements, such as the biparietal diameter (BPD) and transcerebellar diameter (TCD), are important indicators for evaluating GA during second and third trimesters of pregnancy^5, 6^. Increase in atrial diameter (AD), especially moderate-to-severe dilatation, may indicate abnormal fetal brain development^7^. Therefore, in addition to qualitative assessment, quantitative assessment of suspected abnormal brain structures is crucial for prognosis assessment and prenatal counseling^4, 8^.

In clinical practice, these measurements are obtained manually by radiologists on magnetic resonance images (MRI) of specific orientations and slices, which are highly dependent on professional knowledge and clinical experience. Additionally, manual measurements are time-consuming and laborious, and may introduce intra- and inter-observer variability, which can influence the accurate judgment of fetal brain development, leading to inappropriate pregnancy management. Therefore, automating fetal brain biometry through advanced artificial intelligence technology to provide faster, more accurate and reproducible measurements is necessary for the improvement of workflow and accuracy of prenatal diagnosis.

We planned to establish an image segmentation-based artificial intelligence-assisted method by utilizing 268 coronal fetal brain MRI data to achieve automatic linear measurements of the fetal brain, and analyze normal fetal brain growth and development trajectory.

## Materials and methods

### Study population

The fetal brain MRI images collected at West China Second University Hospital, Sichuan University between January, 1, 2020 and November, 31, 2021 were retrospectively analyzed. Inclusion criteria were as follows: (1) prenatal MRI is required for suspected fetal abnormalities using clinic or ultrasound diagnosis; (2) singleton pregnancy with no abnormalities seen in the diagnosis by fetal brain MRI; and (3) MRI image quality was excellent, the scanning position was symmetric, and fetal movement was slight, which did not affect the diagnosis and measurement. Exclusion criteria were as follows: (1) ultrasound showing head circumference (HC), abdominal circumference or femur length exceeding 3 standard deviation (SD) of the fetus at similar GA; (2) pregnant women with fetal chromosomal abnormalities, maternal infections or adverse pregnancy history; and (3) pregnant women with irregular menstruation, inaccurate last menstrual date.

This study was approved by West China Second University Hospital, Sichuan University’s Institutional Review Board. Data collection and analysis were performed in accordance with relevant guidelines and regulations. The informed consent was waived by West China Second University Hospital, Sichuan University’s Institutional Review Board due to retrospective nature of study.

### Fetal MRI protocol

Magnetic resonance imaging scanners included Siemens MAGNETOM Skyra3.0T and Philips Achieva 1.5T Nova Dual MRI system with a phased-array body coil and a large field of view. Maternal sedatives or contrast agents were not administered. Scanning sequences included T_2_ weighted imaging (T_2_WI) single-shot fast spin echo (SSFSE), balanced steady-state free precession (BSSFP), T_1_ weighted imaging (T_1_WI), and diffusion weighted imaging (DWI). The scanning orientations included the axial, coronal, and sagittal planes of the fetal brain and maternal uterus for the SSFSE and BSSFSP sequences. The coronal scan of the fetal brain was perpendicular to the interhemispheric fissure and parallel to the brainstem. The number of slices depended on the size of the fetal brain, and the scanning time was not more than 30 min (range: 13.32–29.27 min, mean: 21.2 min, SD: 3.92 min). The coronal images of the T_2_WI SSFSE sequence of the fetal brain were selected as the training and testing sets, and the coronal and sagittal images of the maternal uterus of the T_2_WI SSFSE sequence were used to determine the left and right body positions of the fetus. The scanning sequence and parameters used in the automatic method are listed in Table 1.

**Table 1:** Sequence parameters of coronal fetal brain MRI used for automatic method.

Vendor	System	Sequence	Cases	TE (ms)	TR (ms)	Thickness (mm)	Gap	Matrix	SAR (W/Kg)	FOV	Flip angle
Siemens	MAGNETOM Skyra3.0T	HASTE	139	80	1800	3–5	0	320 × 320	≤ 2.0	350 × 350	140
Philips	Achieva 1.5T	TSE-SSH	129	80	1300	3–5				300 × 300	90

TE, echo time; TR, repetition time; SAR, specific absorption ratio, FOV, field of view.

### Data annotation

The original data were annotated by four professional radiologists on LIFEX-7.1.1, a freeware for radiomic feature calculation (https://www.lifexsoft.org/), to manually delineate the cerebrum, lateral ventricles, and cerebellum on all slices. The anatomical boundaries of each structure were as follows:

Cerebrum: Delineated along the outer edge of the cerebral cortex, including the frontal, parietal, occipital, and temporal lobes. The cavum septum pellucidum and ventricular system were excluded. The cerebrum and brainstem are bounded by a horizontal line between the cerebral peduncles and thalamus.

Cerebellum: Delineated by the cerebellar borders, including the cerebellar peduncles but excluding the fourth ventricle.

Lateral ventricles: The left and right lateral ventricles were drawn separately, excluding the third ventricle.

All annotation results were checked and corrected by a senior radiologist. (see Supplementary Information for annotation example).

### Method

The automatic methothe maximum distance above the Sylvian Fissured of fetal brain MRI biometry, combined with deep and machine learning methods, is divided into two steps: segmentation and measurement, which automatically measure the cerebral biparietal diameter (CBPD), TCD, left and right atrial diameters (LAD/RAD).

1. Deep learning-based segmentation

First, a deep learning network, nnU-Net^9^, which is widely used in biomedical image segmentation, was used to segment the coronal images of the fetal brain in all slices into four parts: cerebrum, cerebellum, left and right lateral ventricles.

Five-fold cross-validation was used to verify the performance of the model. The specific steps were as follows: data of 268 samples were randomly divided into five parts, one part was selected randomly as the validation set each time, and the other four parts were used as the training set for model training. When one round of training was completed, another part that was not repeated in the last round was selected randomly as the validation set, and the remaining four parts were used to train the model. This process was repeated five times to obtain the prediction results for all the 268 samples. The network training used the Adam optimizer. The learning rate was set to 1e−3, loss function was a combination of dice loss and cross entropy loss to improve training stability and segmentation accuracy, and the training epoch was 200. The patch size was set to 320 × 320, and batch size was 31.

2. Machine learning based linear measurements

After segmentation, CBPD, TCD, LAD and RAD were computed respectively with machine learning methods. The measurement region was determined, and the corresponding fetal brain biometric parameters were calculated according to the manual method clinically used^10^, as well as the anatomical characteristics of the cerebrum, cerebellum, and lateral ventricles:

- Measurements computation of CBPD and TCD.

**Figure 1:**
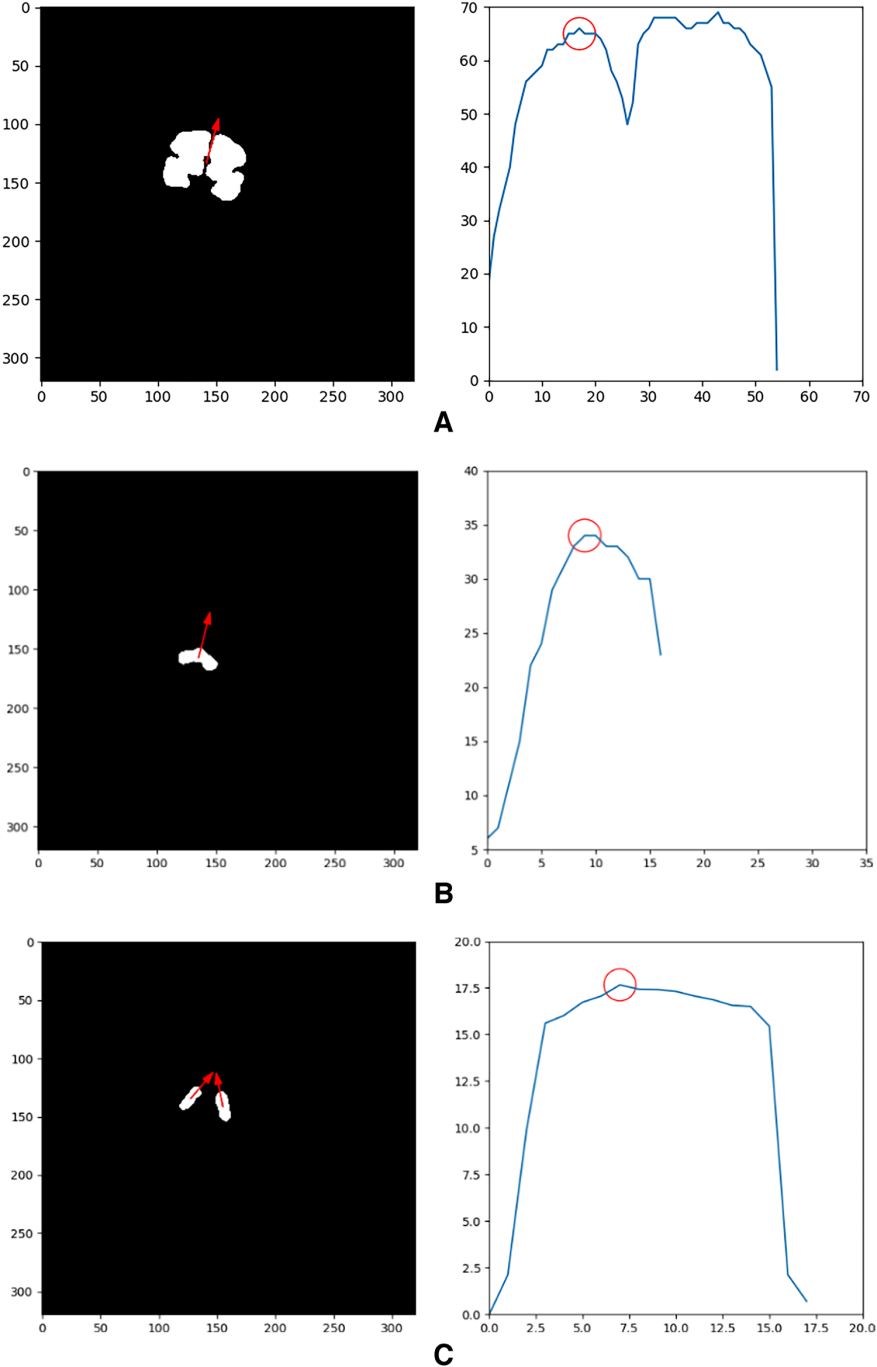
Linear measurements of CBPD, TCD, LAD, and RAD. (**A**–**C**) Are CBPD, TCD, and LAD, RAD, respectively. The red arrow in the left image represents the orientation of the segmentation mask, the right is the curve of the distances perpendicular to the contour orientation, and the red circle represents the measured value.

First, the maximal bounding rectangle based on segmentation mask was found by iterating through all the slices. Second, the orientation of the contour was determined (inferior and superior in anatomy, as shown by the red arrow in the Fig. 1A, B), and then calculate all the distances perpendicular to the main direction.

Combined with clinical knowledge, the maximum distance above the Sylvian Fissure (at approximately 1/2 from the top of the cerebrum) is CBPD, and TCD is the maximum distance calculated.

(b) Measurements computation of LAD and RAD.

First, we used a template to locate the slice at the level of the atria. Second, the orientations of the long axis of the right and left lateral ventricles were determined separately (red arrows in the Fig. 1C), and finally the distances perpendicular to the long axis were calculated and the maximum values were taken as LAD and RAD.

Two-dimensional manual measurements (i.e., CBPD, TCD, LAD, and RAD) were obtained manually by a fetal neuroradiologist (> 30 years of experience) on the original coronal MR of the fetal brain on the picture archiving and communication system, an image workstation for daily use by radiologists to view, diagnose, and analyze images. The specific measurement methods are as follows:

Cerebral biparietal diameter is the maximum transverse diameter of the brain parenchyma above the Sylvian fissure in all the slices (usually at the level of the temporal horns of the lateral ventricles). Similarly, TCD is the maximum transverse diameter of all slices containing the cerebellum (usually at the level of the atria), while LAD/RAD is the inner diameter of the atrium perpendicular to the center of its axis.

### Statistical analysis

Continuous variables were tested for normal distribution. If the data were normally distributed, they were expressed as mean ± SD, otherwise, the median and range were used. The significance level was set to α = 0.05, and all statistical analyses were performed using GraphPad Prism 9.3.0.

#### Automatic measurement method evaluation

Dice similarity coefficient (DSC) was calculated for segmentation task, which measures the similarity between manually segmented region and automatically segmented region.

With manual measurements as the “ground truth”, Pearson’s correlation coefficient (R) was used to assess the correlation between automatic and manual measurements, and the Bland–Altman plots were used to evaluate the agreement between the two methods. The mean difference (MD) between the manual and computerized measurements was used to quantitatively assess the errors. Correlation analysis of errors and GA was used to assess the robustness of the automatic method.

#### Inter-observer agreements

A subgroup of fetuses was selected at random to test inter-observer reproducibility for manual measurements between two expert radiologists. Intra-class correlation (ICC) analysis and difference were used to evaluate the agreement between radiologists.

#### Analysis of automatic measurements and its relationship with GA

Correlation and regression analyses were performed between the obtained automatic biometric parameters and GA. The decision coefficient (R^2^) was used to select the best-fitting model.

## Results

### Patients characteristics

**Figure 2:**
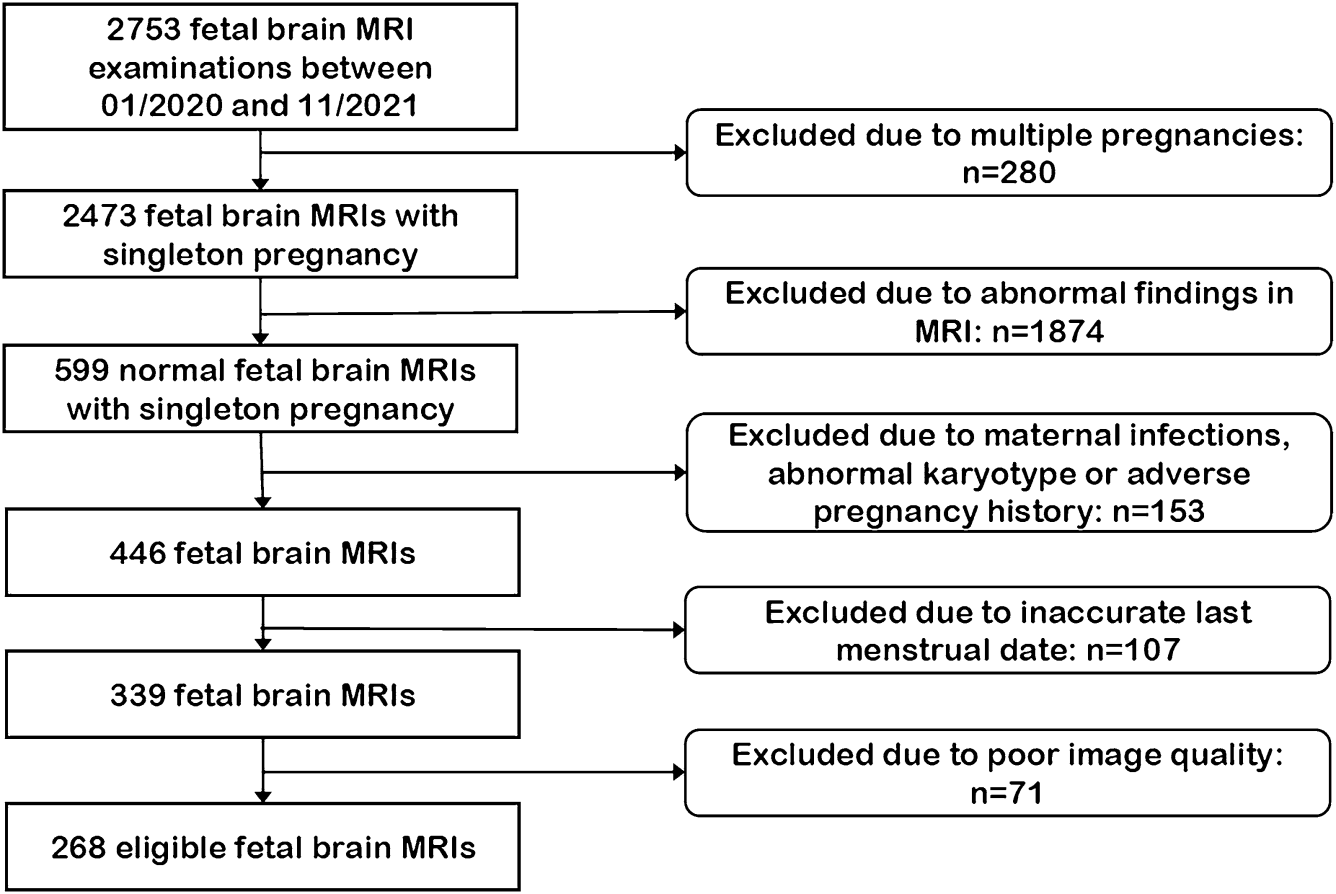
Flowchart summarizing the inclusion and exclusion criteria.

**Figure 3:**
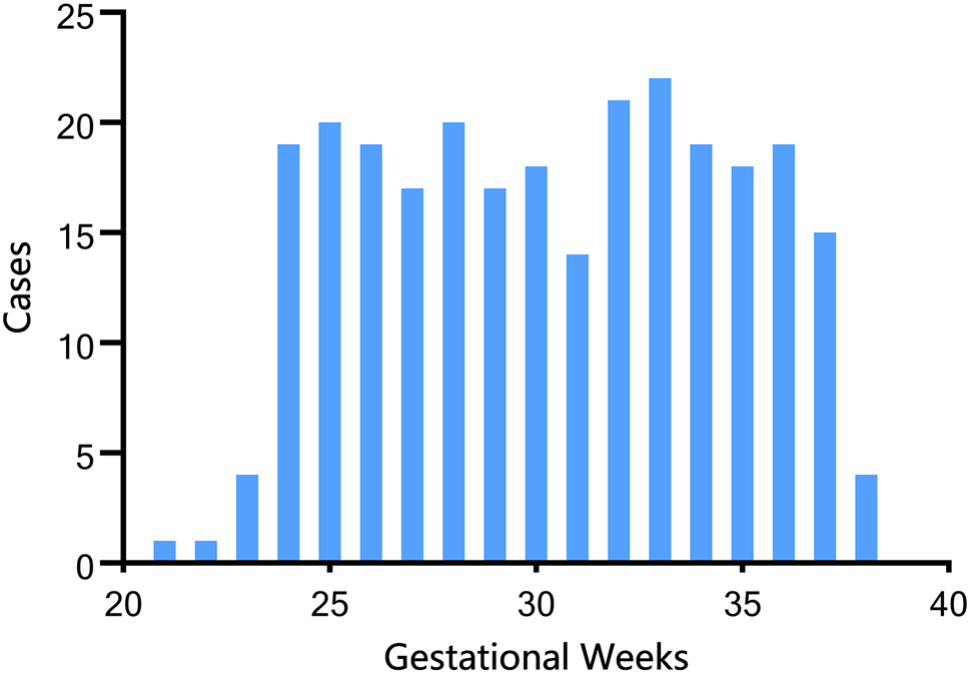
Gestational age distribution of 268 patients.

A total of 5360 coronal brain T_2_WI SSFSE images of 268 fetuses were included according to the inclusion and exclusion criteria (Fig. 2). And the indications of fetal brain MRI are shown in Table 2. The median GA at the time of examination was 30.86 weeks, and the range was 21.29 ~ 38.71 weeks. Fetuses no more than 24 GA accounted for 9.3%. The distribution of GA is shown in Fig. 3.

**Table 2:** The indications of fetal brain MRI.

Type			n (%)
Ultrasound	Decreased cavum septum pellucidum(< 1 cm)		32 (11.9)
	Discordance between fetal size and GA (< ± 3 SDs)		
		Decreased BPD or HC	110 (41.0)
		Increased BPD or HC	29 (10.8)
	Increased cerebellomedullary cistern(> 1 cm)		15 (5.6)
	Unclear vermis		5 (1.9)
	Cystic structures		
		choroid plexus cyst	9 (3.4)
		cavum velum interpositum/cavum Vergae	10 (3.7)
		subependymalcysts	8 (3.0)
		Blake′s pouch	4 (1.5)
	Agenesis of the corpus callosum		1 (0.4)
	Ventriculomegaly (within 10–12 mm)		25 (9.3)
	Subependymal hemorrhage		3 (1.1)
Clinic	Old age pregnancy (> 35 years old)		17 (6.3)

GA, gestational age; BPD, biparietal diameter; HC, head circumference.

### Evaluation of segmentation

**Figure 4:**
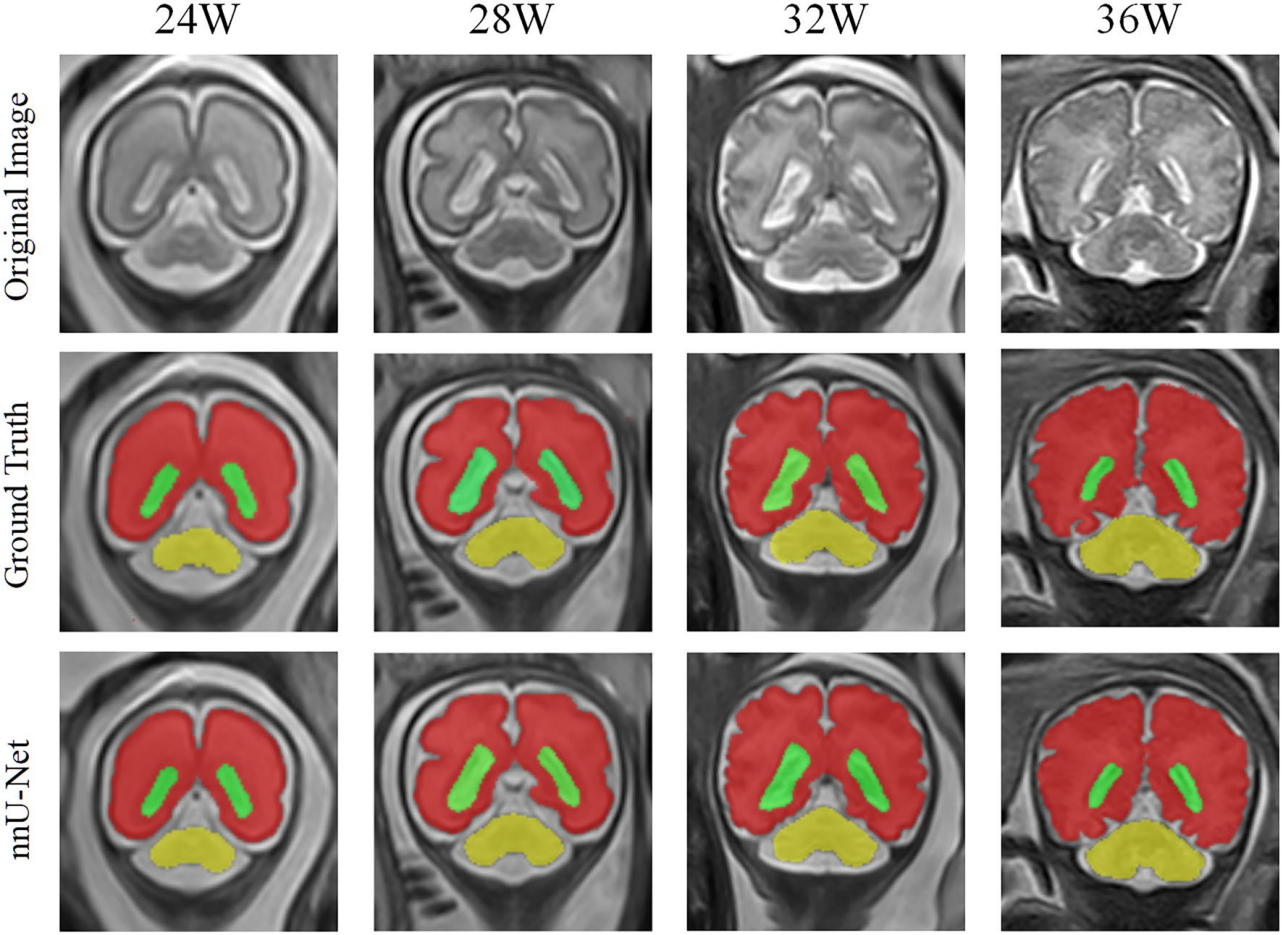
Comparison of segmentation results at different gestational weeks between manual delineation by lIFEx and Auto-segmentation using nnU-Net.

Table 3 shows the average Dice values of the five-fold cross-validation experiments, The nnU-Net based automatic segmentation has achieved high accuracy on the cross-validation of our dataset, which has a mean Dice of 0.9547 ± 0.0015, 0.9116 ± 0.0031, and 0.8507 ± 0.0352 for cerebrum, cerebellum, and lateral ventricles, respectively. The comparison of manual delineation and automatic segmentation at different GA is displayed in Fig. 4.

**Table 3:** The Dice similarity coefficient of automatic segmentation based on nnU-Net.

	Fold 1	Fold 2	Fold 3	Fold 4	Fold 5	Average (SD)
Cerebrum	0.9552	0.9538	0.9571	0.9534	0.9538	0.9547 (0.0015)
Cerebellum	0.9110	0.9090	0.9168	0.9115	0.9096	0.9116 (0.0031)
Lateral ventricles	0.8631	0.8649	0.9115	0.8739	0.8664	0.8507 (0.0352)

### Automatic biometric measurements evaluation

The Pearson correlation coefficients between the manual and computerized measurements of the CBPD, TCD, LAD and RAD were 0.977, 0.990, 0.817 and 0.797, respectively. The MDs are − 2.405 mm, − 0.008 mm, − 0.33 mm and 0.213 mm, respectively.

**Figure 5:**
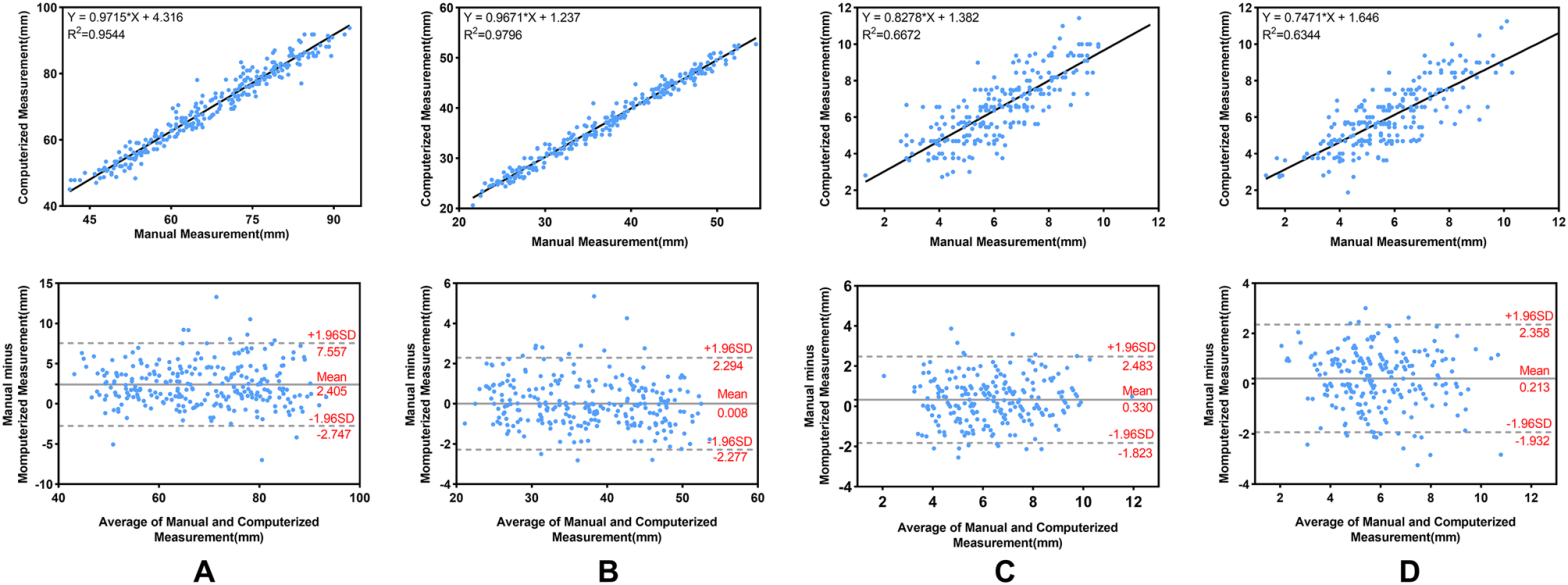
Scatter and Bland–Altman plots between computerized and manual measurements. (**A**–**D**) Are CBPD, TCD, LAD, and RAD, respectively. CBPD, cerebral biparietal diameter; TCD, transcerebellar diameter; LAD, left atrial diameter; RAD, right atrial diameter.

The Bland–Altman plot shows the difference between the automatic computerized and manual measurements. The dotted line in the middle represents the average difference between the manual and computerized measurements, and the upper and lower dotted lines represent a 95% agreement limit, respectively. A vast majority of measurement points for all metrics were within the 95% range (Fig. 5).

**Figure 6:**
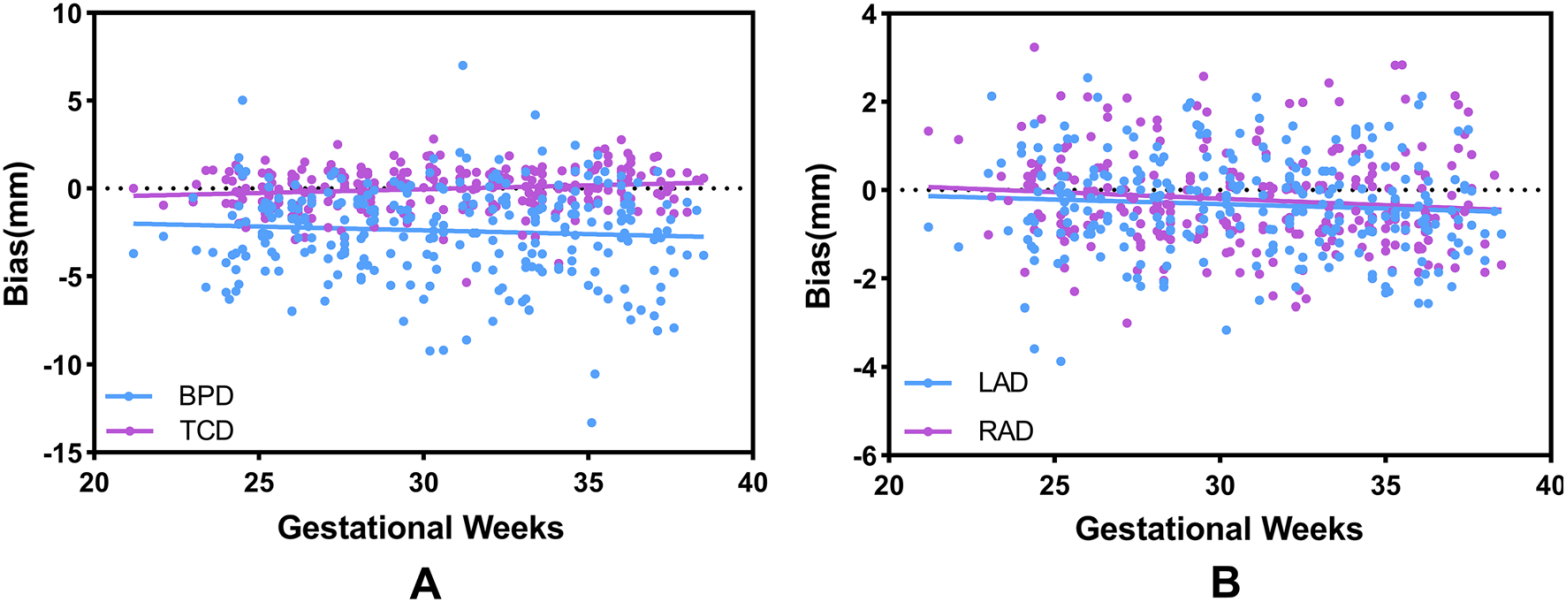
Scatter plot between automatic measurement error and GA. (**A**) CBPD and TCD; (**B**) LAD and RAD. CBPD, cerebral biparietal diameter; TCD, transcerebellar diameter; LAD, left atrial diameter; RAD, right atrial diameter.

The relationship between automatic measurement error and GA is shown in Fig. 6. The bias of all measurement indicators did not vary significantly throughout pregnancy, and was not significantly correlated with GA (correlation coefficients are as follows: CBPD = − 0.069, TCD = 0.156, LAD = − 0.079, RAD = − 0.115; P > 0.05). The predicted differences in bilateral AD were almost similar with GA, and there was no difference between the sides (AD: t = 1.969, p = 0.178).

### Inter-observer agreements for manual measurements

Inter-observer agreements for manual measurements between two radiologists for the subgroup of 52 fetuses that were used for evaluation. The results are shown in Table 4.

**Table 4:** Inter-observer reproducibility of manual measurements between two expert radiologists.

	CBPD	TCD	LAD	RAD
ICC	0.994	0.997	0.945	0.922
Difference	0.176	0.026	0.025	0.020

### Growth analysis between measurement parameters and GA

Based on the established automatic method, the mean, SD, and interquartile range of CBPD, TCD, LAD, and RAD for each GA are calculated (see Supplementary Information). Owing to the small sample size of 21–23 and 38 GA (≤ 4), only the detailed measurement results (n = 258) of 24–37 GA are shown here.

**Figure 7:**
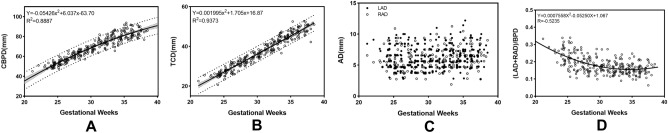
Nonlinear regression analysis between biometric parameters and GA based on automated measurements. (**A**–**C**) are CBPD, TCD, and AD in sequence, (**D**) changes in (LAD + RAD)/CBPD index with gestational age. All the 2D measurements were strongly correlated with GA except for AD, which was not significantly correlated with GA, and the quadratic regression was the best fit. The solid line represents the best fit curve. The light gray area is the 95% confidence interval, and the upper and lower dashed lines are the 95% prediction interval.

For all 2D measurements, quadratic regression showed the best goodness of fit. With the exception of lateral ventricle width, all brain biometric measurements increased with GA (Fig. 7A–C), and had strong correlations with GA (correlation coefficients of CBPD and TCD were 0.9399, 0.9681, respectively). The AD remained stable throughout the second and third trimesters, and was not significantly correlated with GA (the correlation coefficients of LAD and RAD with GA were 0.068 and 0.1602, respectively). The average LAD was 6.44 ± 1.83 mm, and the RAD was 5.88 ± 1.65 mm, there was a slight difference in the width of the bilateral atria (p < 0.001), and the difference between the two sides was about 0.56 mm.

Regression analysis between bilateral atrium diameters and CBPD is shown in Fig. 7D. There was a moderate correlation between bilateral AD and CBPD, with a correlation coefficient of − 0.5235 (p < 0.0001), indicating that the fetal brain grows with increasing GA while the size of the lateral ventricles remains relatively constant.

**Figure 8:**
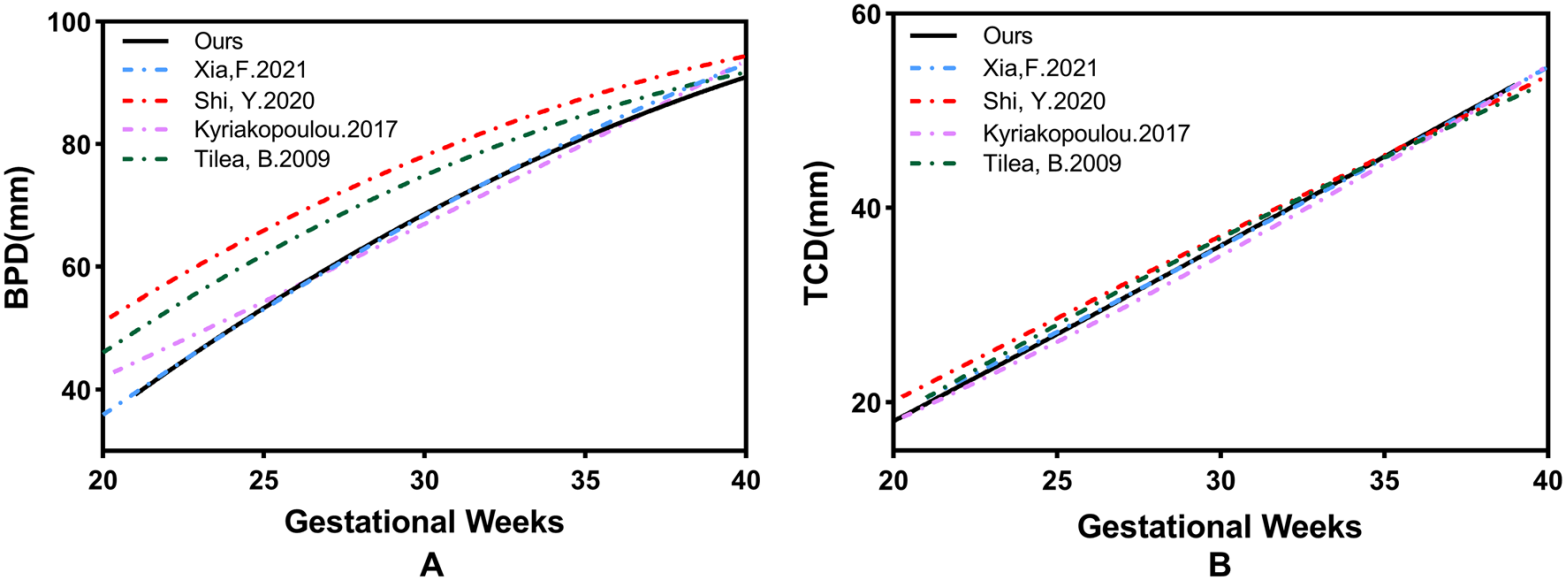
Changes in linear measurements of normal fetal brains with gestational age based on automated measurements compared with previous studies. (**A**, **B**) are BPD, and TCD, respectively.

Figure 8 also shows the change in CBPD and TCD with GA from our measurements and previous studies^6, 11–16^. All the results showed that these biometrics increased with GA, but there were slight differences in the reported values ​​and growth trajectories.

## Discussion

### Synthesis of results and comparison with existing literature

In this study, using 268 coronal MRI data of fetal brain, we established a combined method for accurate and rapid automatic measurements of CBPD, TCD, and AD through segmentation. Additionally, we provided reference biometry and growth trajectories of normal fetus at 24–37 weeks.

By utilizing the advanced deep learning segmentation methods, nnU-Net, we have achieved precise segmentation of the cerebrum, cerebellum and lateral ventricles in fetal MRI, which determines the accuracy of quantitative measurement to a great extent. And based on the accurate segmentation, we developed an automatic method for the computation of coronal biometric parameters with machine learning algorithms. The experiments showed that there was a good correlation between computerized and manual measurements (Fig. 5). For the 2D linear measurements, CBPD and TCD achieved high correlation coefficients of 0.977 and 0.990, respectively, and MD of − 2.405 mm and − 0.008 mm, respectively. In comparison, LAD and RAD had lower correlation coefficients, 0.817 and 0.797, respectively, but still significantly correlated with manual measurement (p < 0.0001). Quantitative error analysis showed that their MDs were all < 0.5 mm. The Bland–Altman analysis (Fig. 5) showed that a high degree of agreement was obtained between the computer-predicted and manual measurements for all linear measurements. Additionally, we also analyzed the relationship between MD and GA (as shown in Fig. 6). Although the fetal brain shape, volume, etc. change significantly with increase in GA, the established automatic method can still remain robust. Errors with manual measurements did not vary significantly throughout pregnancy. The above results demonstrate that the automated method achieves human-level performance and can cope with high variability in inputs such as multiple scanners, multiple field strengths, and multiple GA.

Because ultrasound has been the first choice for prenatal screening of fetuses for decades, MRI-based studies of the automatic measurement of fetal brain biometry have only recently begun to emerge. Avisdris et al.^17^ used convolutional neural networks to achieve automatic measurement of bone BPD, CBPD, and TCD in fetal brain MRI for the first time. Although we both adopt deep learning-based segmentation followed by linear measurements computation, there are still significant differences between our studies. In addition to the different deep learning networks chosen, the biggest one is that their research requires reference slice selection and only segments the two selected slices, while ours segment all the slices of the fetal brain. This is very important because by segmenting total brain, we can compute the CBPD and TCD of all slices to select the maximum value, which is more accurate and in line with the clinical definitions than the results obtained by reference slice. As for biometric parameters measurement, our method achieved comparable performance for the CBPD and TCD measurements (mean absolute difference, 2.25 mm vs. 1.21 mm, 1.24 mm vs. 1.26 mm; 95% confidence interval [CI], 10.3 mm vs. 7.9 mm and, 4.6 mm vs. 6.5 mm, respectively). Furthermore, we performed 2D quantitative analysis of the lateral ventricles. Accurate measurement of AD is important because small technical differences can lead to false-positive or false-negative results. Experiments showed that, for both lateral ventricles, our automatic method obtained a measurement error of < 0.5 mm, which is in good agreement with manual measurement.

### Clinical implications

The morphology of fetal brain changes dramatically throughout pregnancy, which increases the difficulty of interpretation of fetal MRI radiologists. However, measurements of the diameters and volumes of the fetal brain increase with GA. When there are abnormalities in brain development, such as malformation, mass lesions, or obstruction, the measurements also change accordingly. Therefore, assessment of fetal brain development using biometric measurements is a simple and effective method.

As the brain parenchyma matures, the shape of the lateral ventricles changes accordingly and the volume decreases, but the AD remains constant in the second and third trimesters, with a normal value of < 10 mm. When the AD is ≥ 10 mm, it is called ventriculomegaly, which is the most common indication for referral to MRI by ultrasound, accounting for 40% of fetal central nervous system MRI indications^8, 18, 19^. The etiology of ventriculomegaly and the degree of dilatation determine its neurodevelopmental prognosis. Severe ventriculomegaly (AD > 15 mm) has a poor long-term prognosis, with approximately 40% having severe disability and 18% having mild-to-moderate disability. When combined with other intracranial or extracranial malformations, the morbidity to mortality ratio ranges from 6% and rose to 56%^20, 21^. Therefore, assessment of lateral ventricular size is an important part of prenatal imaging.

According to the recommendations of the International Society of Ultrasound in Obstetrics and Gynecology guidelines (2020)^3^, prenatal examination of the fetal head should routinely measure the BPD, HC, TCD and AD etc. As an important auxiliary imaging method of ultrasound, MRI can be in large-scale, multi-planar, and multi-parameter with brilliant soft tissue resolution. Additionally, MRI is not affected by the amount of amniotic fluid, thickness of subcutaneous fat, whether the fetal head enters the pelvis, and degree of ossification, making it possible to precisely measure the anatomy of the brain^22^. The general linear measurements (such as HC, BPD, etc.) established in ultrasound can reflect the size of the fetal head, but the inconsistent growth rate and characteristics of trade-offs of various parts of the fetal brain cause abnormalities in fetuses with normal skull size. Consequently, more comprehensive biometric measurements of the fetal brain by MRI (such as bone and cerebral biparietal diameters, extracerebral space, and brain volume) can improve the accuracy of fetal prenatal diagnosis and reduce misdiagnosis.

In this study, with the established automatic method, we obtained a large sample (n = 268) of 2D and 3D biometric data of normal fetal brain. Based on the automatic measurement data, we displayed the growth and development of fetal brain in the second and third trimesters in multiple directions, including the mean, SD, interquartile range, growth curve, 95% prediction interval, and the relationship between different parameters. Additionally, we compared the results with those of previous studies to verify the reliability and validity of the data.

Analyses showed that the CBPD, and TCD increased with GA in a secondary growth pattern, consistent with other MRI studies^6, 10–16^. For CBPD, the variation between our reference value and growth trajectory and the results reported by other studies is relatively large, which may be related to the different measurement methods used by different researchers, including the report Shi et al.^6^ in which they measured the maximum transverse diameter of the skull in an “outer-inner” way in the axial section; that is, the measured value includes not only the brain parenchyma but also the cerebrospinal fluid space and part of the skull, which explains why the reported BPD are overall higher than those in other studies. Quantitative analysis of brain volume showed that, our results are almost consistent with those of other previous studies, except for Jarvis^14^, because the reported brain parenchyma volume included both cerebral hemispheres, cerebellum, and brainstem. Compared with other biometric parameters, TCD was less susceptible with minimal variation between studies. The growth trajectories almost completely overlapped, and maintained high consistency even in the third trimester. This may indicate that TCD maintains a higher accuracy as pregnancy progresses, which has important implications for the determination of GA in the third trimester.

Although the shape of the lateral ventricle changes significantly with GA, AD remains stable in the second and third trimesters, and is not significantly correlated with GA. The average value was approximately 6.2 mm, which is consistent with the conclusions of previous studies^23–25^. The ratio of AD to CBPD decreased with an increase in GA, which also reflects the fact that the fetal brain volume increases, while the ventricular size remains relatively constant during pregnancy. Machado-Rivas et al. ^26^ reported the characteristic of asymmetry of fetal brain development, in which there was leftward asymmetry for lateral ventricles, and in our study, the AD also showed significant differences (P < 0.0001), wherein the LAD is about 0.56 mm wider than the RAD.

### Strengths and limitations

Our study has two major strengths. First, the data size and GA included are the largest in similar studies, which also includes a combination of multi-manufacturer scanners and multi-field strength. The diversity of the dataset makes this automatic method more applicable. Second, we obtained a large sample of biometric measurements of normal fetal brain based on the proposed method and performed multifaceted analysis of biometric parameters to characterize the normal growth of fetal brain.

Our study has several limitations. First, the distribution of GA in our dataset was unbalanced, especially the images in the early second trimester and late third trimester (≤ 23 and ≥ 38 GA), which is mainly due to the inherent disadvantage of retrospective studies. This is because in current clinical practice, fetal MRI is recommended at 20 GA and beyond. Second, we only evaluated normal fetal brains, and the applicability of this method in the presence of central nervous system malformations has not been tested. Third, we only trained and tested SSFSE sequence images, and it remains to be carefully evaluated whether BSSFP, the other most commonly used sequence in fetal MRI, can achieve similar results. Additionally, for the analysis of cerebral growth, our sample was selected from those suspected to be abnormal during prenatal ultrasound, but ultimately diagnosed as normal, and no follow-up after birth was performed to confirm that the data obtained were indeed from normal fetuses. However, a multicenter prospective study by Griffiths et al.^27^ showed that the diagnostic accuracy of fetal MRI was 93% with very low false-positive and false-negative rates. Limited by the sample size of the corresponding GA, reference values of fetal cerebral biometric parameters < 23 and ≥ 38 GA are not listed because of lack of accuracy. Though we are committed to establishing a comprehensive automatic tool for biometric parameters of fetal brain MRI for clinical use, coronal measurements are only our preliminary results, and measurements in other two orientations, such as HC in axial images and measurements of corpus callosum, vermis, and brainstem in sagittal images, are currently under investigation. In the future, we will conduct multicenter experiments, accumulate larger datasets, further evaluate the performance of this automatic method on more diverse datasets, and perform prospective studies to establish comprehensive 2D and 3D reference values for fetal brain MRI at complete GA.

## Conclusion

We proposed an image segmentation-based artificial intelligence-assisted measurement method that can achieve fast and accurate automatic measurement of biometric parameters of fetal brain MRI through two steps of segmentation and measurement. We also provided reference values of fetal growth, growth curves, and a 95% prediction interval. Therefore, biometric data based on automatic methods can assist clinicians in evaluating fetal development, thereby facilitating prenatal diagnosis and improving clinical work efficiency.

### Supplementary Information

Supplementary Information.

## Data Availability

The datasets used and analyzed in this study are available from the corresponding author upon reasonable request.
