# Supplementary material for: Automatic biometry of fetal brain MRIs using deep and machine learning techniques
Source: Sci Rep. 2023 Oct 19;13:17860. doi: 10.1038/s41598-023-43867-4 (PMC10587162; doi:10.1038/s41598-023-43867-4)

Supplemental information

Automatic Biometry of Fetal Brain MRIs Using Deep and Machine Learning Techniques

Jiayan She; Haiying Huang; Zhijun Ye; Wei Huang; Yan Sun; Chuan Liu; Weilin Yang; Jiaxi Wang; Pengfei Ye; Lei Zhang; Gang Ning

Supplemental information

Table of contents

1. Automatic results of linear measurements 2

2. Annotation example 4

Automatic results

1.1 Automatic results of CBPD for 258 fetuses at 24–37GA

	GA	Cases	Mean(mm)	SD (mm)	25%	50%	75%
**CBPD**	24	19	51.23	2.51	49.22	50.91	53.44
	25	20	53.49	3.36	51.40	52.50	56.21
	26	19	56.49	3.33	54.46	56.00	57.09
	27	17	61.11	2.60	59.06	60.95	63.05
	28	20	63.47	2.89	60.99	64.49	64.92
	29	17	65.61	3.60	63.52	66.36	68.44
	30	18	68.74	3.12	66.67	68.03	70.80
	31	14	73.65	4.42	71.53	74.11	76.02
	32	21	74.37	4.14	72.19	73.64	76.88
	33	22	78.96	5.86	76.09	79.55	83.20
	34	19	78.58	4.42	75.97	78.18	81.09
	35	18	84.33	5.36	79.24	84.22	88.13
	36	19	83.71	4.50	80.47	82.00	86.87
	37	15	84.82	4.44	81.24	85.31	88.36

1.2 Automatic results of TCD for 258 fetuses at 24–37GA

	GA	Cases	Mean(mm)	SD (mm)	25%	50%	75%
**TCD**	24	19	25.59	1.56	25.16	25.66	26.21
	25	20	27.27	1.52	26.53	27.66	28.13
	26	19	29.18	2.22	27.09	30.00	30.94
	27	17	31.58	1.56	30.94	31.44	32.73
	28	20	32.70	1.58	31.88	31.94	33.88
	29	17	34.79	1.93	33.72	34.62	36.41
	30	18	36.05	1.44	34.69	36.10	37.00
	31	14	38.94	1.79	38.00	38.76	40.74
	32	21	39.77	2.21	38.10	38.44	41.82
	33	22	43.21	2.81	41.86	43.06	45.00
	34	19	44.24	1.64	43.72	44.41	45.00
	35	18	46.65	2.41	44.77	46.88	47.95
	36	19	47.38	1.86	46.36	46.88	48.85
	37	15	49.10	2.27	47.54	48.57	50.91

Automatic results

1.3 Automatic results of LAD for 258 fetuses at 24–37GA

	GA	Cases	Mean(mm)	SD (mm)	25%	50%	75%
**LAD**	24	19	6.82	1.73	5.67	6.56	8.03
	25	20	6.24	2.00	4.47	6.56	8.18
	26	19	6.19	2.34	4.62	6.00	8.22
	27	17	6.19	1.78	4.95	5.45	7.50
	28	20	6.20	1.52	5.47	5.86	7.18
	29	17	5.89	1.80	4.36	5.63	6.80
	30	18	6.04	1.66	4.71	5.86	7.27
	31	14	6.66	2.06	5.42	5.86	7.43
	32	21	5.84	1.57	4.69	5.00	7.50
	33	22	6.92	1.78	5.50	6.95	8.47
	34	19	5.90	1.42	4.84	5.71	6.56
	35	18	7.13	2.30	5.63	6.92	8.20
	36	19	6.99	1.60	6.18	6.56	7.75
	37	15	6.87	1.92	5.99	7.00	7.84
**Total**		258	6.44	1.83	4.93	6.36	7.50

1.4 Automatic results of RAD for 258 fetuses at 24–37GA

	GA	Cases	Mean(mm)	SD (mm)	25%	50%	75%
**RAD**	24	19	5.71	1.50	4.72	5.63	6.56
	25	20	5.75	2.09	4.69	5.05	6.56
	26	19	5.09	1.72	3.88	4.69	5.63
	27	17	5.71	1.29	4.77	5.54	6.56
	28	20	5.07	1.63	3.75	4.69	5.63
	29	17	5.84	1.79	4.69	5.54	6.75
	30	18	5.82	1.29	5.11	5.63	6.50
	31	14	6.00	1.64	5.15	5.67	6.27
	32	21	6.24	1.63	5.45	6.00	7.50
	33	22	6.28	2.07	4.82	5.99	7.59
	34	19	6.10	1.10	5.54	5.71	6.79
	35	18	6.15	1.96	4.71	6.46	7.20
	36	19	6.19	1.38	5.23	5.71	6.92
	37	15	6.11	1.73	5.11	6.36	6.78
**Total**		258	5.88	1.65	4.69	5.63	6.67

Annotation Example

This is an annotation example of fetal cerebrum, cerebellum and lateral ventricles in coronal plane.

Software: LIFEX-7.1.1
Annotation style: slice by slice
Gestational age of the fetus: 33 weeks

Labels:

- Cerebrum
- Cerebellum
- Left lateral ventricle
- Right lateral ventricle

Annotation Example

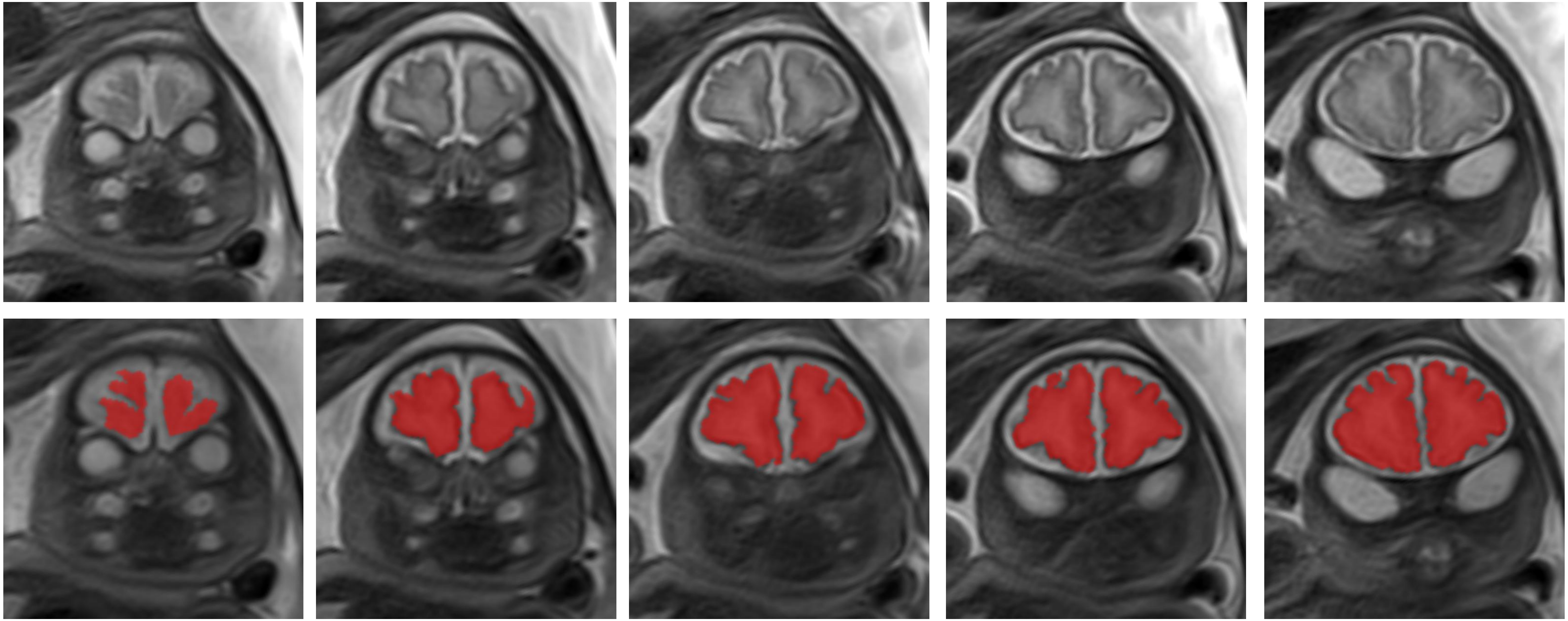

Annotation Example

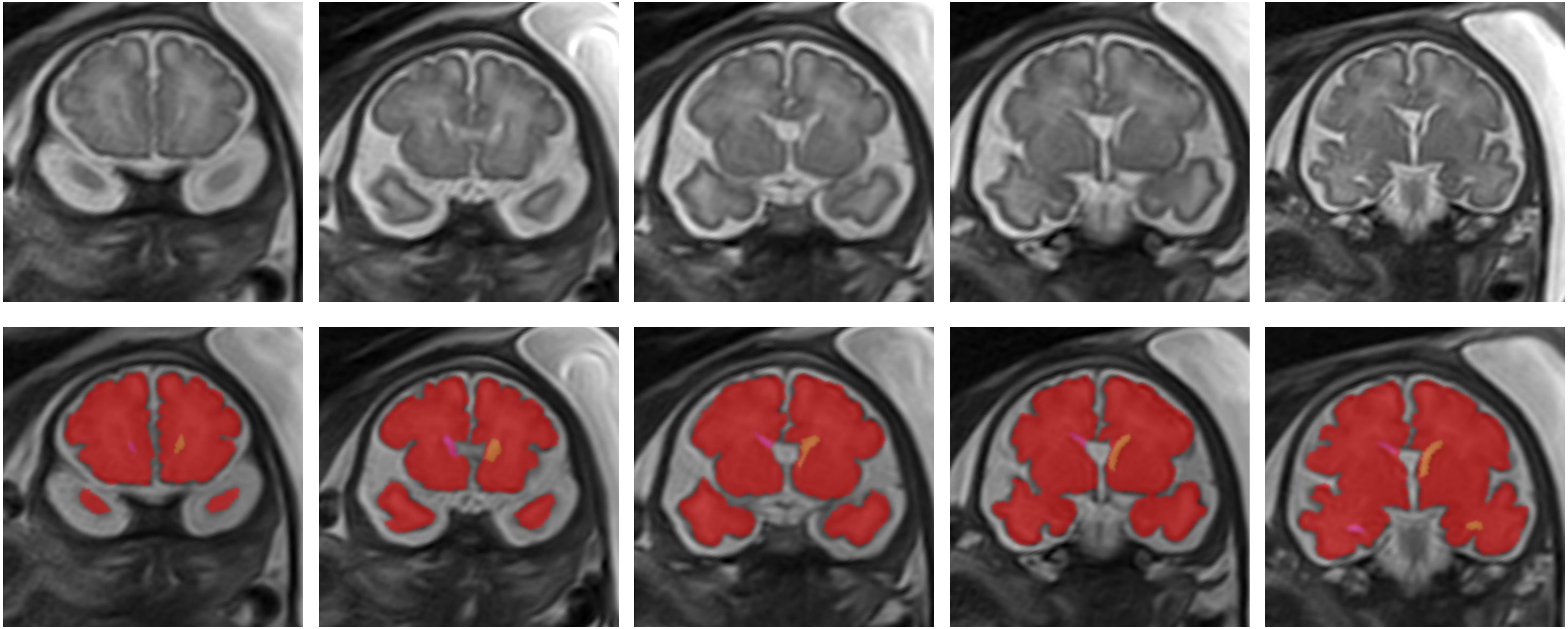

Annotation Example

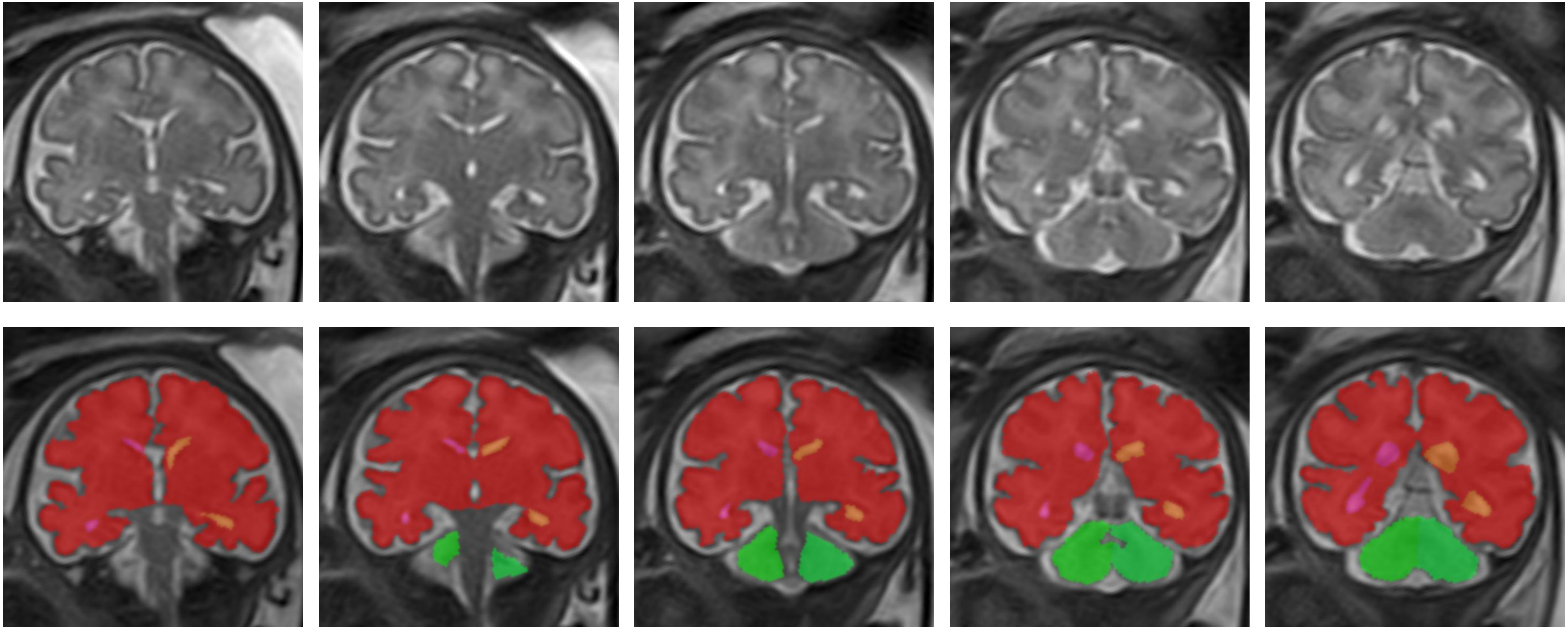

Annotation Example

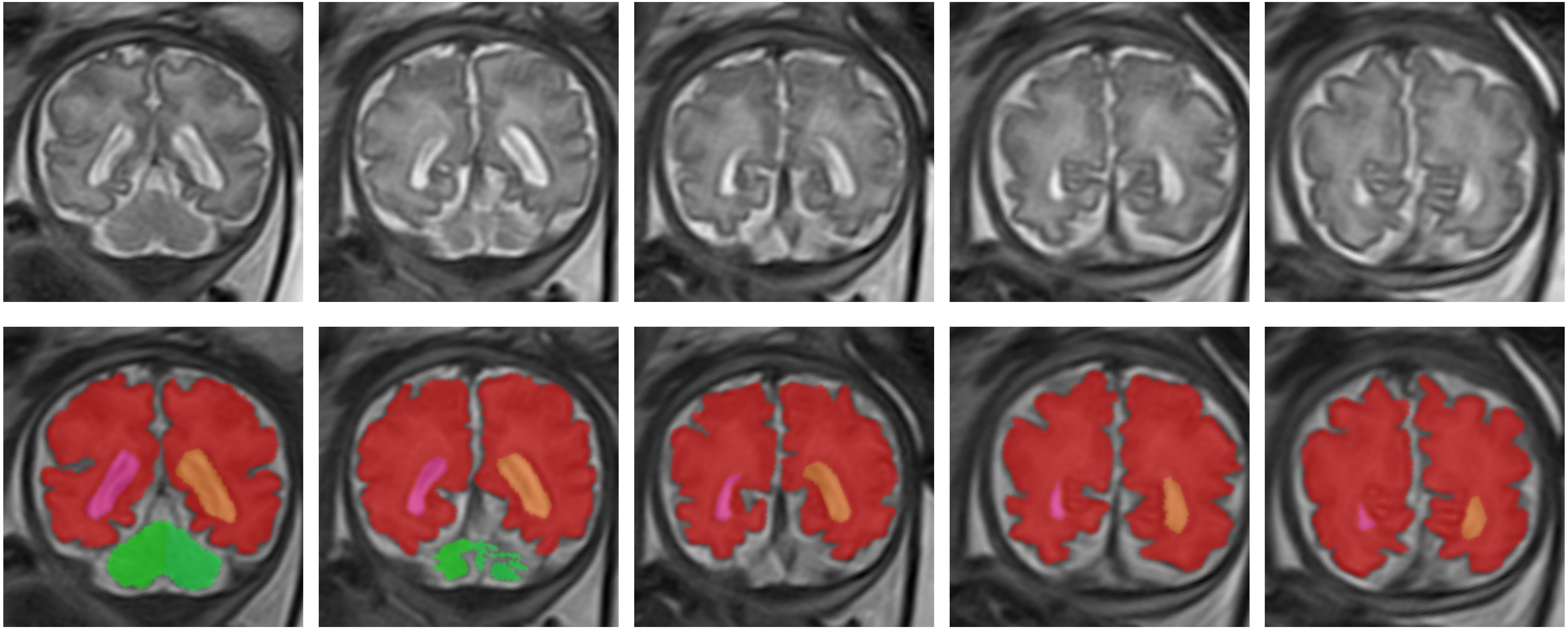

Annotation Example

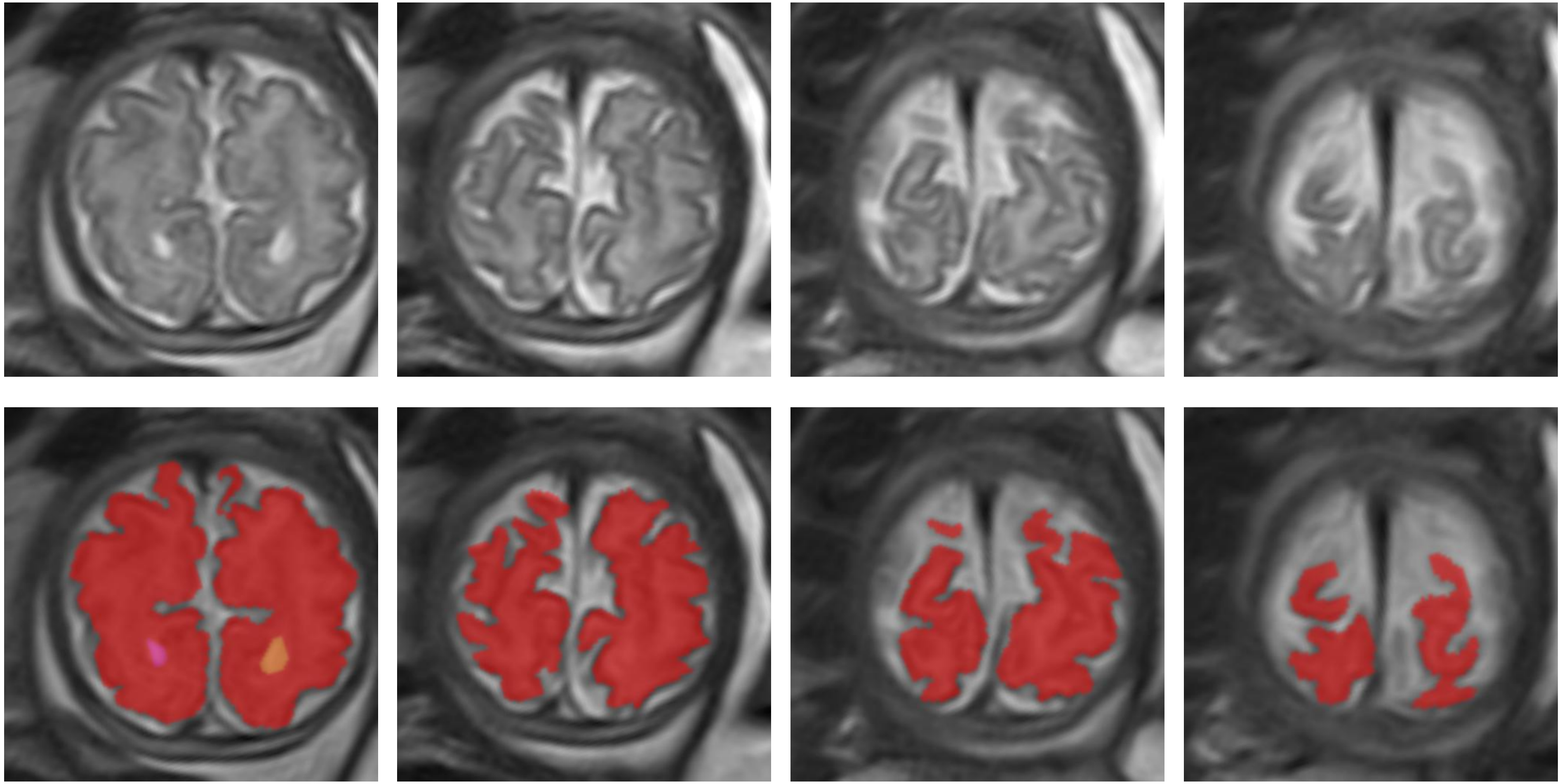

Supplement: Supplementary file 1 — Supplementary Information. [file 41598_2023_43867_MOESM1_ESM.pdf]
